# Assessing the impact of mutations found in next generation sequencing data over human signaling pathways

**DOI:** 10.1093/nar/gkv349

**Published:** 2015-04-16

**Authors:** Rosa D. Hernansaiz-Ballesteros, Francisco Salavert, Patricia Sebastián-León, Alejandro Alemán, Ignacio Medina, Joaquín Dopazo

**Affiliations:** 1 Computational Genomics Department, Centro de Investigación Príncipe Felipe (CIPF), Valencia, 46012, Spain; 2 Bioinformatics of Rare Diseases (BIER), CIBER de Enfermedades Raras (CIBERER), Valencia, 46012, Spain; 3 HPC Services, University of Cambridge, Cambridge, CB3 0RB, UK; 4 Functional Genomics Node, (INB) at CIPF, Valencia, 45012, Spain

## Abstract

Modern sequencing technologies produce increasingly detailed data on genomic variation. However, conventional methods for relating either individual variants or mutated genes to phenotypes present known limitations given the complex, multigenic nature of many diseases or traits. Here we present PATHiVar, a web-based tool that integrates genomic variation data with gene expression tissue information. PATHiVar constitutes a new generation of genomic data analysis methods that allow studying variants found in next generation sequencing experiment in the context of signaling pathways. Simple Boolean models of pathways provide detailed descriptions of the impact of mutations in cell functionality so as, recurrences in functionality failures can easily be related to diseases, even if they are produced by mutations in different genes. Patterns of changes in signal transmission circuits, often unpredictable from individual genes mutated, correspond to patterns of affected functionalities that can be related to complex traits such as disease progression, drug response, etc. PATHiVar is available at: http://pathivar.babelomics.org.

## INTRODUCTION

Whole exome and genome sequencing are becoming mainstream methodologies in the discovery of new disease genes. While finding disease genes is relatively easy in Mendelian pathologies or *de novo* highly penetrant syndromes, this task can become extraordinarily difficult in the case of common diseases (1). Because of their multigenic nature, most complex diseases are better understood as failures of functional modules caused by different combinations of mutated genes rather than by unique mutation(s) in one single gene. This idea of a modular nature of human genetic diseases (2), that explains phenomena such as epistasis (3), incomplete penetrance, non-reproducibility of biomarkers (4), etc. can be helpful for finding disease genes (5), drug targets (6), new-generation mechanism-based biomarkers (7), etc. in the context of known functional modules. Particularly, signaling pathways are functional modules that include a representation of the knowledge available on the consequences that the combined effect of protein activities has over the cell functionality in response to different stimulus.

Recently, some methods based on signaling pathways aim to discover activation of sub-networks within them (8–10). Others, more specific, focus on the estimation of the activity of stimulus-response signaling circuits from gene expression data (11–13). Stimulus-response signaling circuit activities can be inferred from gene expression measurements and provide a rich-informative type of biomarkers which can be further used for predictive purposes (7). Despite the obvious potential of pathways as conceptual tools to understand the effects of gene mutations over cell signaling, the lack of user-friendly applications for this purpose drastically limits the current application of these methods.

Here we present a new web server, PATHiVAR, which can be used to easily infer mutations that are expected to have relevant consequences for cell functionality, because they affect genes belonging to signaling pathways. These consequences can be further related to complex phenotypes, such as disease, drug response, etc. Since different tissues have different patterns of gene expression, the predicted consequences will be tissue-specific. PATHiVAR uses a simple Boolean model to infer the probabilities of signal transmission in human signaling pathways from any receptor protein to any final effector protein. The Boolean model used here is a simplification derived from the statistical model used in PATHiWAYS (12,13). The model uses known gene expression values, taken from two popular curated gene expression repositories (Human Protein Atlas (14) and Expression Atlas (15)), to derive individual probabilities of gene activation that are combined according to the signaling circuit wiring to infer what we consider the unperturbed pattern of signal transmission across all the selected pathways in the selected tissue. Then, standard variant calling format (VCF) input files, containing the variants detected in individuals, are scanned for variants in the protein coding genes that compose the pathways. The deleteriousness of such variants is evaluated according to the variant consequence types, along with thresholds based on extensively used pathogenicity indexes that can be configured by the user. Finally, the information on protein functionality is integrated in the model by removing all the proteins impaired by deleterious mutations. The model is recalculated for the perturbed (mutated) system. The comparison between the unperturbed and the perturbed models provides relevant clues on the impact of variants over cell signaling.

PATHiVAR has been used during the last year in the context of the Spanish Network for Research Rare Diseases (CIBERER; http://www.ciberer.es) in the last step of gene prioritization of the candidate genes produced by the BiERapp tool (16) (http://bierapp.babelomics.org).

PATHiVAR aims to provide clues to understand how the interactions among the proteins that compose signaling pathways account for cell functionalities and how perturbations of such functionalities relate to complex phenotypes such as diseases. To our knowledge, there is no other similar tool available. PATHiVAR can be found at: http://pathivar.babelomics.org.

## EVALUATION OF THE DELETERIOUSNESS OF A MUTATION IN THE CONTEXT OF SIGNALING

### Modeling the human *signalome*

A Boolean model is used to model the probabilities of signal transduction from receptor to effector proteins across 26 human KEGG pathways (17) from the general categories Environmental Information Processing and Cellular Processes, which include relevant processes and systems such as Signal Transduction (*ERBB, WNT, NOTCH, JAK-STAT, calcium, VEGF, HEDGEHOG* and *mTOR signaling pathways*), Cell Growth and Death (*apoptosis* and *p53 signaling pathway*), Cell Communication (*GAP junction* and *tight junction*), Signaling Molecules and Interaction (*neuroactive ligand-receptor interaction, cell adhesion molecules, cytokine-cytokine receptor interaction* and *EMC-receptor interaction*), Endocrine System (*insulin signaling pathway, adipocytokine signaling pathway, PPAR signaling pathway, GnRH signaling pathway* and *melanogenesis*) and Immune System (*toll-like receptor signaling pathway, B cell receptor signaling pathway, T cell receptor signaling pathway, Fc epsilon RI signaling pathway, antigen processing and presentation*, and *chemokine signaling pathway*).

Signaling circuits are defined as the sub-pathways (within the pathways) that transmit signals from a receptor node, which receives the stimulus to an effector node that triggers the response. Such circuits can include bi- or multi-furcations and typically consist of nodes that activate other nodes but they can also contain nodes that inhibit the activity of other nodes. Nodes can be composed of one or more proteins. In the Boolean model, gene expression is taken as a proxy of the presence of a protein in the pathway (8,9,12,13,18). Probabilities of 1 (presence) or 0 (absence) are assigned to any of the proteins in the pathway according to the gene expression reported for the tissue of interest in the database selected for the analysis. A total of 48 tissues from the Human Protein Atlas (14) and 66 from the Expression Atlas (15) databases are available. Node probabilities are computed as: (i) the product of their constituent protein probabilities, if the node is a protein complex or (ii) as the maximum of the values of the protein probabilities, if these are alternative (12,13). Once the probabilities for each node in the circuit have been assigned for a particular tissue, a simple probabilistic product from the receptor node to the effector node, across all the connecting nodes in the pathway, can be used to model the probability of signal transmission (12,13). We call *unperturbed signaling pattern* to the probabilities of signal transmission calculated for all the stimulus-response circuits across all the selected pathways in the particular analyzed tissue.

The models here cover an ample range of tissues compiles in the Human Protein Atlas (14) and the Expression Atlas (15) databases. However, some genes might have peculiar expression values that could be not properly or accurately represented in the databases or present atypical expression behaviors in certain conditions. These scenarios can be modeled by using the *Additional gene list* box, which allows specifying values for a list of genes that overwrite the values reported in the database. In addition, a complete list of user-defined gene expression values can be provided through the button *Custom* for conditions not represented in the tissue databases used.

### Predicting the deleterious effect of a mutation

Not all every variant found is expected to have a predictable impact on signal transmission. Consequence types (see http://www.ensembl.org/info/genome/variation/predicted_data.html) describe a primary classification of possible variant effects. Only a subset of consequence types is compatible with variation that affects protein coding genes. These are: *exon_variant, intron_variant, synonymous_codon, coding_sequence_variant, non_synonymous_codon, splice_region_variant, splice_acceptor_variant, splice_donor_variant, stop_gained, stop_lost, 5_prime_UTR_variant, 3_prime_UTR_variant, 5KB_upstream_variant, 5KB_downstream_variant*.Consequence types are taken from CellBase (19).

Typically, stop loss, stop gain and splicing disrupting variants are considered damaging mutations. The impact and damaging effect of non-synonymous variants depend on the type of amino acid change and can be predicted by computing SIFT (20) and PolyPhen (21) damage scores. Outside the coding regions this prediction can be extended using the phastCons (22) conservation score. By default, variants with a phastCons conservation score higher than 200, a SIFT score lower than 0.05 or a PolyPhen score higher than 0.95 are considered to have a damaging effect on the affected protein. SIFT, PolyPhen and phastCons values are taken from CellBase (19) as well, through the VARIANT (23) tool (see database version annotations at https://github.com/opencb/cellbase/wiki/Data-Sources-and-Species). The default choice is: *non_synonymous_codon, stop_gained* and *stop_lost* consequence types, with SIFT and PolyPhen thresholds of 0.05 and 0.95, respectively, which corresponds to the more obvious scenario of deleterious, loss-of-function variants.

### Predicting the effect of a deleterious mutation over signaling

Once the *signalome* has been modeled and the *unperturbed signaling pattern* in any tissue has been derived, the prediction of the effect of deleterious variants affecting to pathway proteins is straightforward. PATHiVAR removes from the model those proteins harboring deleterious mutations (as defined by the user) that impairs its proper functionality and recalculates the probability of signal transmission across all the stimulus-response circuits of the pathways studied in the selected tissue again, obtaining thus the *perturbed signaling pattern*.

### Web interface functionality and data management

PATHiVAR can be directly used in anonymous mode (*Run* item in the main menu), and therefore all the uploaded data and the results obtained (and not saved in the user's terminal) will be lost at the end of the session. The program can also be used in ‘registered user’ mode. Registration is free and the options are the same, the only difference is that registered users can maintain their data and results in the PATHiVAR workspace with a limit of 10 GB.

**Figure 1.**
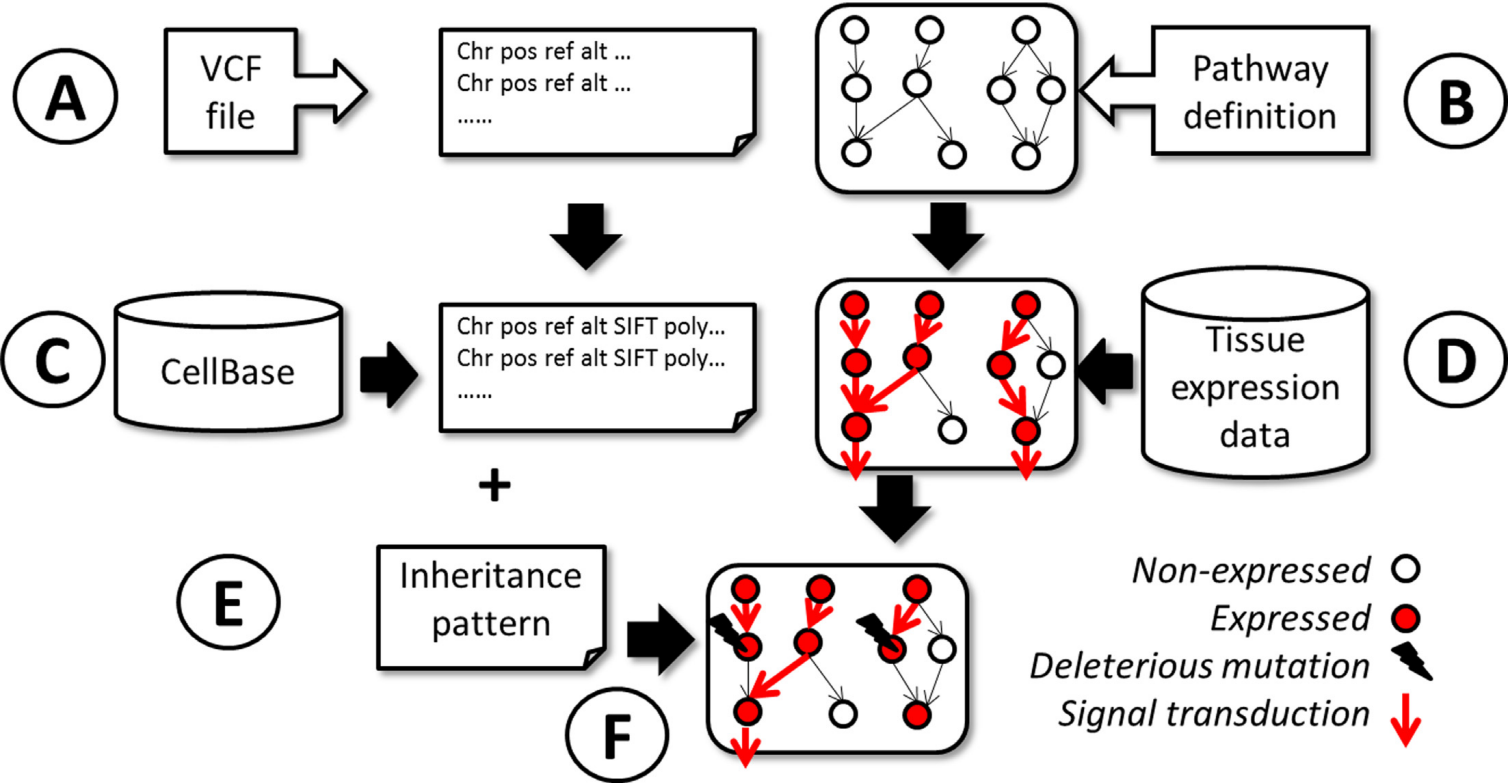
Schema of the analysis of impact of mutations over signaling pathways. (**A**) VCF files are uploaded in the system. (**B**) KEGG pathway definitions are selected. (**C**) VCF files are annotated using information contained in CellBase. Consequence type, SIFT, PolyPhen and phastCons indexes are associated to each variant position in the VCF. (**D**) Tissue is selected (or user-defined pattern of gene presence/absence is uploaded) and the unperturbed map of signal transduction (corresponding to the functional genes in the tissue) is deduced from the presence/absence of the genes in the pathway. (**E**) Depending on the inheritance pattern (dominant/recessiv/compound heterozygote) the expressed but damaged proteins are removed from the model and the net signal transduction is inferred again which produces (**F**) the perturbed map of signal transduction. The differences between the unperturbed (D) and the perturbed (F) signal transduction maps are reported by PathiVar. The bottom right of the figure show the symbols used to denote expressed and non-expressed genes, genes harboring deleterious mutations and the interactions that produce signal transduction.

Running the program invokes the main form, where data can be uploaded and parameters for the analysis are provided. The upload option (*Select VCF file* option) of the form brings about the *STUDIES* workspace, where VCF data files reside, or where they can be uploaded from the local computer. If the file selected is a multisample VCF, the specific sample to be analyzed can be selected with the *Sample name from VCF* option (see Figure 1A). The pathways to be analyzed are selected (Figure 1B). The VCF to be analyzed is annotated with consequence type, SIFT, PolyPhen and phastCons values, taken from CellBase (19) (see Figure 1C). The user selects the tissue of interest (with the possibility of changing some gene expression values if necessary, through the *Additional gene list* box) or provides a complete list of user-defined gene expression through the button *Custom* that will be used for the model to derive the unperturbed system (Figure 1D). Then, the inheritance pattern of the sought mutations can be specified (dominant or recessive). Compound heterozygosity (having simultaneously two different recessive alleles at a particular locus) is supported. Subsequent options allow choosing the consequence types considered in the study and setting thresholds for the pathogenicity indexes. This information will be used to remove genes affected by deleterious mutations from the model and thus deriving the perturbed system (Figure 1E and F).

Once the analysis parameters have been defined, a name can be assigned to the job and then the algorithm can be run (*Launch job* button). Once the job is launched, it appears in the job list in running state. When the job is finished and the result is available, the job appears in the finished status with a check symbol.

### Systems biology inspired interpretation of the results

**Figure 2.**
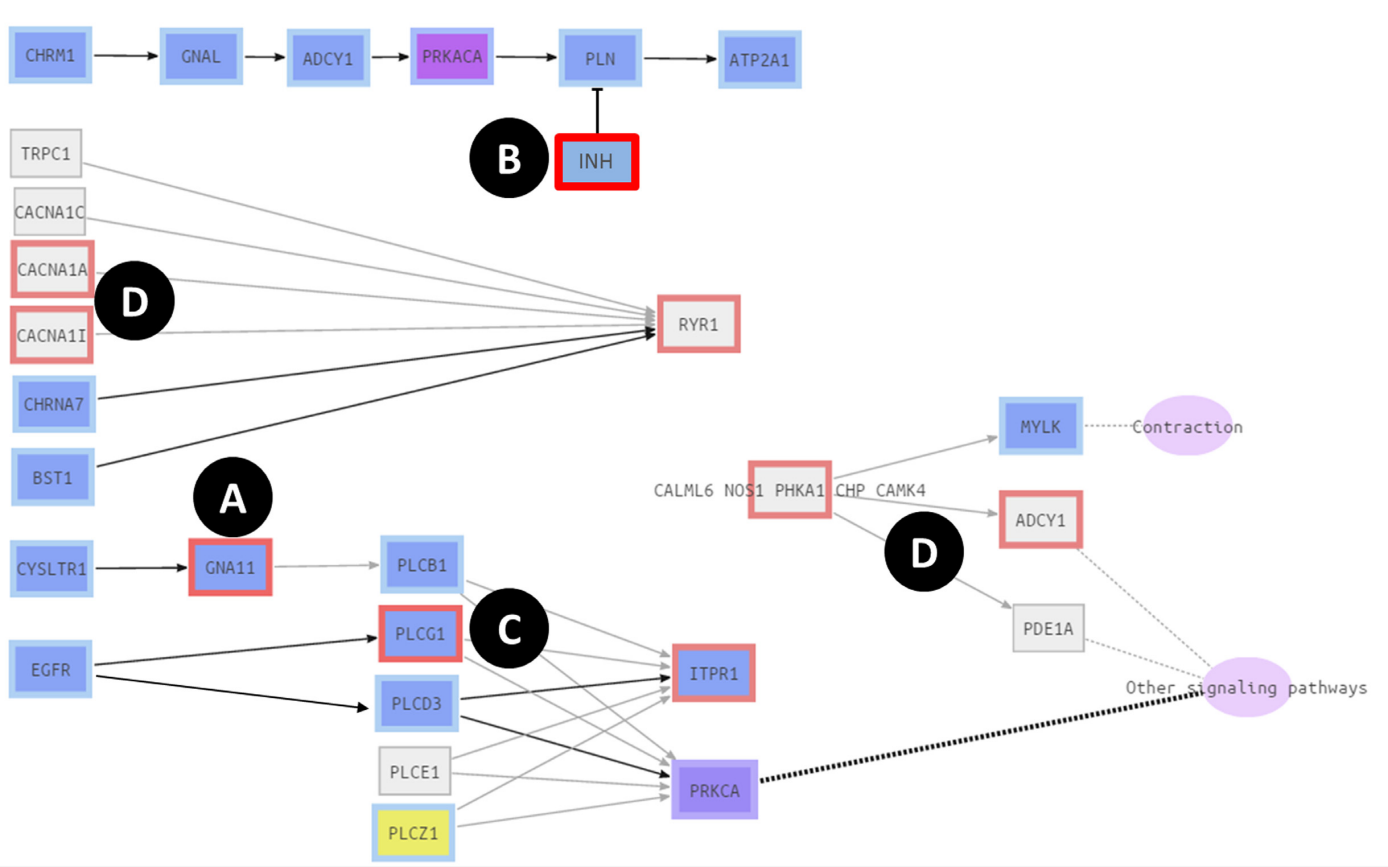
Output of a fictitious combination of gene expression and mutations in version of the *Calcium signaling pathway* modified for illustrating the examples. The figure illustrates the possible effects due to the combination of gene expression and gene loss of function within the topology of a pathway. In the figure, blue background indicates gene expression, while gray background means no expression in the tissue studied. Yellow background means unknown expression in the tissue. Black arrow indicates signal transmission whereas gray arrow means no signal is transmitted. (**A**) signal deactivation: the gene *GNA11* is expressed but harbors a deleterious mutation and the signal does not flux downstream; (**B**) signal activation: the repressor *INH* harbors a deleterious mutation and therefore cannot inhibit *PLN* and the signal flux, that would be interrupted here with a functional protein, is activated instead; (**C**) neutral effect: *PLCG1* with a deleterious mutation does not transmit the signal, however, The signal is transmitted anyway from EGFR to both, *ITPR1* and *PRKCA*, through the protein *PLCD3*, because the receptor-effector signaling circuit is internally redundant; (**D**) neutral effect: several examples show how mutations affect to genes that are not expressed in the tissue studied (*CACNA1A, CACNA1I, RYR1*, etc.) and consequently have no effect in the particular tissue of study.

The resulting effect of the combination of gene expression and gene product integrity, when interpreted over the system defined by topology of the pathways, can be diverse and often have unexpected consequences. Figure 2 represents different scenarios that can be found when complex signaling circuits are analyzed under a systems biology perspective and include: (i) signal deactivations, when the impaired proteins interrupts the signal flux in the receptor-effector signaling circuit in which they are included; (ii) signal activations, when the affected protein is a repressor that was short-circuiting the circuit; (iii) neutral effects, when a receptor-effector signaling circuit is internally redundant and the signal can reach the effector protein using an alternative unaffected branch. This third possibility is quite interesting because the study of mutations within a systems perspective allows discarding putative disease-causing genes harboring mutations with apparent deleterious consequences, that the robustness of the pathway turn into innocuous variants; (iv) the integration of both expression and mutation data in the model brings about another possibility, which is typically ignored by conventional models without data integration: deleterious mutations affecting genes that are not expressed in the tissue of interest are irrelevant for signaling in this particular tissue.

The graphical output (Figure 2) represents the pathways analyzed, highlighting the possible ways by which the signal is transmitted from receptor proteins to the corresponding effector proteins. Disruptions in the signal flux can be easily attributed to particular deleterious mutations found in the VCF file. Results are displayed in an advanced interactive visual framework, CellMaps (https://github.com/opencb/cell-maps/wik), which provides a graphical output in which aspect, colors and shapes of the components of the pathway can easily be reconfigured to produce camera-ready figures. In addition to the graphical output, each pathway displays a table which lists the affected circuits with relevant information, including receptor and effector proteins, the activation status and the particular cell functionality triggered by the circuit. A table summarizing the results obtained for all the selected pathways can also be downloaded.

## DISCUSSION

Conventional analysis of genomic data seeks to relate gene expression (24) or mutations (25) to disease or complex phenotypes. However, such relationships are difficult to find and often lack reproducibility (4,26). It is believed that the integration of both types of data (27): gene activity (expression data) and gene functionality (affected or not by deleterious mutations) can be helpful for disease gene prioritization. However, data integration by itself does not capture the complex network of molecular interactions that configure the cellular functionality (28). From a systems biology perspective (29), diseases can be interpreted as the failure of cellular functionalities represented by functional modules, such as pathways (2). Therefore, it is quite likely that different individuals sharing a disease do not show common mutations (or mutations in common genes), but rather have a common cell functionality affected. And this can occur because they present different mutated genes affecting to a common signaling circuit that triggers such functionality (6,7). Here we show how a simple Boolean model, based on KEGG, one of the most popular descriptions of signaling pathways (30), can integrate gene activity (expression) and gene functionality (mutation) in a straightforward manner. In the future we plan to include different signaling pathway descriptions coming from other repositories, such as REACTOME (31), and also allowing users to input their own pathway definitions.

The systems biology perspective, provided by the models of signaling circuits presented here, offers an optimal framework to locate recurrent functional failures that can be better related to diseases or complex traits than the original raw genomic measurement (gene expression or mutation) from which the models were derived.
